# Population pharmacokinetic and exposure-response study of a novel anti-tuberculosis drug to inform its dosage design in phase III clinical trial

**DOI:** 10.1016/j.ejps.2025.107160

**Published:** 2025-09-01

**Authors:** Weijie Kong, Hao Liang, Yi Zhang, Lei Li, Yongguo Li, Xiaoyu Yan, Dongyang Liu

**Affiliations:** a Drug Clinical Trial Center, Peking University Third Hospital, Beijing, PR China; b Department of Nephrology, Peking University Third Hospital, Beijing, PR China; c Shanghai Jiatan Biotech Ltd., a subsidiary of Guangzhou JOYO Pharma Ltd., Shanghai, PR China; d Institute of Medical Innovation, Peking University Third Hospital, Beijing, PR China; e School of Pharmacy, Faculty of Medicine, The Chinese University of Hong Kong, Hong Kong SAR, PR China

**Keywords:** Multi-drug resistance tuberculosis, WX-081, Population pharmacokinetics, Exposureresponse analysis, Dosage design

## Abstract

Although bedaquiline (BDQ) received conditional approval for multi-drug resistance tuberculosis (MDR-TB), a black box warning was added due to QT prolongation risk. WX-081, a promising second-in-class drug that finished phase II clinical trial, exhibited comparable anti-TB activity and better cardiac safety. The accumulation of its active metabolite WX-081-M3 leads to QT prolongation, whereas the relationships between dosage, exposure and response have not been established. Accordingly, the dosage design for phase III study is challenging. Here, the first population pharmacokinetic (PPK) and exposure-response (E-R) analysis were conducted for WX-081. 1610 WX-081 and 1580 WX-081-M3 concentrations were collected from 24 healthy volunteers and 48 tuberculosis patients for PPK study. The pharmacokinetic parameters and sputum culture conversions of 20 MDR-TB patients receiving BDQ and WX-081 were used for E-R analysis. The absorption of WX-081 was well described by a three-compartments transit model, while the distribution and elimination profiles of WX-081 and WX-081-M3 were captured by three- and two-compartments models, respectively. E-R analysis demonstrated that the clinical efficacy of WX-081 is comparable with BDQ and can be evaluated by average concentration at steady state (C_avg,ss_) of WX-081. According to the simulation results of different regimens, the dosage of 450 mg once daily (QD) for 1 week and subsequent 300 mg QD for 1 week followed by 150 mg QD for 22 weeks was recommended considering both efficacy and safety. Our study revealed the PK and efficacy profiles of WX-081 for the first time and proposed a dose optimization strategy to facilitate its clinical development.

## Graphical abstract

**Figure:**
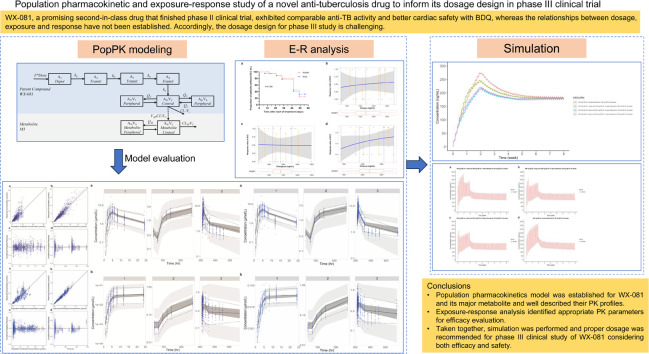

## 1 Introduction

Tuberculosis (TB) is a severe infectious disease caused by *Mycobacterium tuberculosis* that commonly affects lung but can also spread to other parts of the body. TB is among the top 10 causes of death worldwide (Angula et al., 2021), and caused an estimate of 1.6 million deaths and 6.4 million new cases annually (World Health O 2022). Of note, approximately 4 % of new cases are multidrug-resistant tuberculosis (MDR-TB), which is defined as resistant to at least two first-line anti-TB drugs, isoniazid and rifampin (and possibly other drugs). Treatment success rate can regularly achieve at least 85 % for patients with drug susceptible (DS)-TB but dramatically decreases to 60 % for MDR-TB patients (World Health O 2022).

The discovery of novel anti-TB drugs has stagnated for four decades until the approval of bedaquiline (BDQ) (Wong et al., 2013). BDQ, formerly named as TMC207 or R207910, has a novel mode of action that targets mycobacterial adenosine triphosphate (ATP) synthase and thereby disrupts TB energy metabolism (Andries et al., 2005). Though BDQ has demonstrated its anti-TB efficacy and was approved for MDR-TB treatment by the FDA in 2012, a black box warning was added to drug label due to potential safety risk in prolonging QTc interval (Tanneau et al., 2021; Anonymous 2012). Furthermore, increased liver enzyme levels raise additional hepatotoxicity concerns for BDQ usage (Worley and Estrada, 2014). The high price also hinders the worldwide use of BDQ, especially for developing countries where TB is mainly occurring. Thus, there is an urgent need for the development of novel anti-MDR-TB drugs with comparable efficacy, better safety and affordable price.

WX-081 (sudapyridine), a structural analog of BDQ, exhibits similar in vitro activity against DS- and MDR-TB strains in comparison with BDQ (Yao et al., 2022; Huang et al., 2022; Zhu et al., 2022). Comparable efficacy was further validated by acute and chronic mouse tuberculosis models (Yao et al., 2022). WX-081 is metabolized primarily via CYP3A4 to its major metabolite (WX-081-M3) with weaker in vitro anti-TB activity (unpublished data). The hERG IC_50_ of WX-081-M3 (1.89 μM) was slightly higher than that of BDQ-M2 (1.73 μM), the major metabolite of BDQ that is responsible for QT-prolongation risk (Tanneau et al., 2021; Yao et al., 2022). *In vivo* studies showed higher lung tissue concentration, better safety and lower QT interval prolongation risk of WX-081 than those of BDQ (Yao et al., 2022). Furthermore, WX-081 was well tolerated in healthy volunteers (HVs) and TB patients, as demonstrated by phase I (Yu et al., 2024) and phase II studies (unpublished data). Thus, WX-081 was considered as a potential best-in-class candidate for MDR-TB treatment.

Similar to BDQ, WX-081 and WX-081-M3 accumulate as drug dosing due to their long elimination half-lives. The increased drug and metabolite exposure is associated with not only enhanced anti-TB efficacy but also potentially elevated cardiac risk. As a result, to balance the efficacy and safety, the dosage should be designed as the complex combination of a loading dose to obtain early bactericidal activity, and a maintenance dose to maintain antimycobacterial activity as well as to restrict the continued increase of WX-081-M3 plasma concentrations to prevent QT-prolongation toxicity. Therefore, dosage design in phase III study is a major challenge for the clinical development of WX-081.

In this study, we aim to use model-informed drug development strategy to aid the phase III dosage design by (1) establishing the first population pharmacokinetic (PPK) to capture the pharmacokinetic (PK) profiles and identify significant covariates for WX-081 and WX-081-M3; (2) exploring PK parameters that are associated with clinical efficacy by exposure-response (ER) analysis; (3) predicting the PK profiles of WX-081 and WX-081-M3 under different regimen; and (4) designing the dosing regimen by bridging the preclinical and clinical data of WX-081 and BDQ (Madabushi et al., 2022; Rayner et al., 2021; Marshall et al., 2016).

## 2 Methods

### 2.1 Patient population and study design

A total of 24 HVs from phase I trial (study A), 28 DS-TB and 20 MDR-TB patients from phase II trial (study B) were included. A descriptive summary of each study and the detailed demographic characteristics of the whole study population was presented in Table 1.

**Table 1:** Summary of clinical studies used for model development.

Study	**A**	**B**	
**Phase**	**I**	**II**	
Population	HVs	DS-TB	MDR-TB
No. of subjects	24	28	20
WX-081 dose	single dose of 200, 300, or 400 mg for the D1 and QD for D4-D14	150, 300, or 450 mg QD for 14 days	400 mg QD for 14 days followed by 150 mg QD for 6 weeks
No. of PK samples	720	533	357
Duration of sampling	up to 10 days after last dose	up to 2 days after last dose	up to 1 days after last dose
No. of sex	21 Male	21 Male	10 Male
Age (year) *^a^*	28.0 (21.0–37.0)	44.0 (20.0–59.0)	36.5 (20.0–60.0)
Height (cm) *^a^*	168 (156–180)	171 (150–180)	166 (158–178)
Weight (kg) *^a^*	62.6 (52.1–73.5)	58.0 (45.0–77.0)	55.1 (41.0–74.5)
BMI (kg/m^2^) *^a^*	22.2 (19.7–25.4)	20.9 (15.9–26.4)	19.6 (16.0–25.9)
ALT (U/L) *^a^*	12.0 (6.00–31.0)	18.5 (6.00–65.0)	10.0 (4.00–89.0)
AST (U/L) *^a^*	17.0 (12.0–26.0)	16.5 (9.00–104)	15.5 (11.0–77.0)
ALP (U/L) *^a^*	64.5 (39.0–105)	90.0 (53.0–162)	80.5 (47.4–313)
TBIL (μmol/L) *^a^*	11.1 (4.90–23.40)	9.45 (4.10–21.4)	9.46 (4.00–16.3)
CR (μmol/L) *^a^*	70.0 (49.0–85.0)	55.1 (34.1–86.4)	51.1 (40.0–126)

HVs, healthy volunteers; DS-TB, drug-susceptible tuberculosis; MDR-TB, multidrug-resistant tuberculosis; QD, once daily; PK, pharmacokinetics; BMI, body mass index; ALT: alanine transaminase; AST: aspartate transaminase; ALP: alkaline phosphatase; TBIL: total bilirubin; CR: creatinine; *a*, presented as median (range).

Study A is a single-center, randomized, double-blind, placebo-controlled, dose-ascending phase I trial (NCT06117514). Thirty HVs were enrolled and divided into three cohorts. For each cohort, WX-081 or placebo was administered orally under the regimen of 200 mg, 300 mg or 400 mg once daily (QD) on D1 and D3 to D14, respectively. Blood samples were collected prior to and 1, 2, 3, 4, 5, 6, 8, 12, 24, 48, 72, 96, 120, 144, 264 h after the first dose on D1 and the last dose on D14. Study B (NCT04608955) is a multi-center, randomized, parallel, open-label, positive-controlled phase II clinical trial consisting of five cohorts. Three cohorts of DS-TB patients (*n* = 12 for each cohort) received 150 mg, 300 mg and 450 mg WX-081 orally QD for 2 weeks. For the other two cohorts, 40 participants with MDR-TB were randomized to receive either WX-081 (400 mg QD, *n* = 20) or BDQ (400 mg QD, *n* = 20) for two weeks in the first treatment stage. During the second treatment stage, each cohort received WX-081 (150 mg QD) or BDQ (200 mg thrice weekly (TIW)) for six weeks respectively, followed by a four weeks follow-up period. Multi-drug background treatment (MBT) consisted of levofloxacin, isoniazid, cycloserine and pyrazinamide were concomitantly administrated in the second treatment stage. Blood samples (pre-dose and 1, 2, 3, 4, 5, 6, 8, 12, 24 h) were collected in D1 and D14.

Both trials were consistent with the Good Clinical Practice (GCP) standards and received ethical approval from the institutional review board of Shanghai Xuhui Central Hospital (ethical approval number: 2018,048) and Beijing Chest Hospital affiliated to Capital Medical University (ethical approval number: 2020,021), respectively. All participants in the study provided written informed consent.

### 2.2 Bioanalysis

WX-081 and WX-081-M3 plasma concentrations were quantified using a validated liquid chromatography-tandem mass spectrometry (LC-MS/MS) methodology (Yao et al., 2022). The lower limit of quantification (LLOQ) was 10 μg/L and 0.1 μg/L for WX-081 and WX-081-M3, respectively.

### 2.3 Population pharmacokinetic model development

Plasma concentrations of WX-081 and WX-081-M3 from all studies were converted to molar units and used to development PPK model. Nonlinear mixed-effects model was applied to establish PPK model describing typical PK characteristics and variability between or within individuals.

To describe the absorption process of WX-081, both transit compartment model and linear absorption model were evaluated in the structural PPK model. According to the multiphasic distribution of BDQ, two- or three-compartment and one- or two distribution models were used to describe the distribution of WX-081 and WX-081-M3, respectively. Due to unknown parent to metabolite conversion fraction (*F_M_*), both the clearance (*CL_M_*) and volume of distribution of WX-081-M3 (*V_c,M_*) are unidentifiable. As a result, either *F_M_* or *V_c,M_* need to be fixed. Given the similarity in the structural and physio-chemical properties between WX-081 and WX-081-M3, we adopted the assumption that the volume of distribution of WX-081-M3 is identical with that of WX-081.

The stochastic model included between-subject variability (BSV) and residual variability. BSV was modeled in an exponential form (eqs. 1):

(1) $$P_{i}=TVP\times e^{\eta_{i}}$$

where $P_{i}$ and TVP is the *i*th individual and typical value of the parameter, respectively; $\eta_{i}$ is the empirical Bayes estimates of BSV for the *i*th individual that is normally distributed with mean zero and variance ω^2^.

The residual variability was assessed as an error model in a proportional form (eqs.2):

(2) $$C_{ij}=\overset{\wedge }{C_{ij}}\times \;\left(1+\varepsilon_{pro}\right)$$

where $C_{ij}$ and $\overset{\wedge }{C_{ij}}$ is the *i*th observed and predicted concentration for the *j*th individual, respectively; $\varepsilon_{pro}$ is the proportional error component that is normally distributed with mean zero and variance σ^2^.

### 2.4 Covariate model development

Based on a correlation analysis, covariate that correlated with PPK parameters (r^2^>0.3) were selected as candidates for further analysis. Demographic parameters including age, weight, sex, HP (a binary variable defined as HVs or patients), SMOKE (a binary variable defined as whether one person smokes) and the other parameters in relation with liver function including alkaline phosphatase (ALP), aspartate aminotransferase, alanine aminotransferase, total bilirubin and cholesterol, were considered to be candidates. Since both the phase I and phase II clinical study were conducted in the Chinese population, race was not included for covariate evaluation.

The construction of covariate model was conducted by forward inclusion and backward elimination. In forward inclusion step, each candidate covariate was added to the structural model and the corresponding object function value (OFV) was assessed. Covariate models with OFV reduction (∆OFV) >3.84 (*P* < 0.05) were considered to have significant influence on the structural model. The covariate with the largest ∆OFV was added to the structural model and subjected to the next round of evaluation until ∆OFV of all remaining covariates was <3.84. Then, ∆OFV criterion of 6.63 (*P* < 0.01) was used for the backward exclusion, where each covariate included in the forward inclusion step was excluded one by one to calculate corresponding ∆OFV. At last, the final model was established with covariates that passed all screening criteria.

Continuous and categorical covariates were established by power function and linear function (eqs. (3) & 4), respectively:

(3) $$P_{i}=\theta_{1}\times {\left(\frac{Cov_{con}}{Cov_{median}}\right)}^{\theta_{2}}$$

(4) $$P_{i}=\theta_{1}\times \left(1+\theta_{2}\times Cov_{cat}\right)$$

Where P*_i_* is the parameter for the *i*th individual; $Cov_{con}$ and $Cov_{cat}$ is the value of continuous and categorical covariate for the *i*th individual, respectively; $Cov_{median}$ is the median value of continuous covariate; $\theta_{1}$is the typical value for the population parameter; $\theta_{2}$ is the estimated value for the covariate effect.

### 2.5 Model evaluation

Goodness-of-fit (GOF) plots and visual predictive checks (VPCs) were used to evaluate the final PK model. GOF plots described the curves of population predicted (PRED) or individual predicted plasma concentrations (IPRED) versus observed value (DV), as well as and conditional weighted residuals (CWRES) versus PRED or the time after last dose. VPC was used to assess the predictive performance of the final model by simulating 1000 replicates. Due to sparse sampling of WX-081 between D2 and D13, VPC was separated into three periods (D1, D2 to D13, and D14) for better display.

In addition, the relative standard errors (RSEs) of the parameter estimates and the condition number of the model was also considered for the final model. Finally, the results of bootstraps with 500 replicates were used for model assessment.

### 2.6 Model-based simulation

Model-based simulations were carried out using final PPK model to estimate the time-concentration profiles of WX-081 and WX-081-M3 under different regimens. The dosage was listed in Table 3.

### 2.7 Calculation of exposure parameters

The area under the concentration-time curve in 24 h at steady state (AUC_ss_) at D56 was calculated for WX-081 using linear trapezoidal method for ascending and log trapezoidal method for descending concentrations (linear up log down) by non-compartmental analysis (NCA). The following exposure parameters were all derived from the simulation results at steady state using the established PPK model and individual parameters. The average plasma concentration of WX-081 at steady state (C_avg,ss_) was calculated as AUC_ss_ divided by 24 h. The peak (C_max,ss_) and trough concentration of WX-081 at steady state (C_trough,ss_) were also calculated according to the concentration before and after the last dose, respectively. The peak concentration (C_max,WX-081−M3_) at D14 was determined for WX-081-M3.

### 2.8 E-R analysis

E-R was conducted to explore the relationship between sputum culture conversion (SCC) and different exposure parameters (C_avg,ss_, C_max,ss_ and C_trough,ss_). The correlation was measured by logistic regression analysis. Kaplan-Meier plot of SCC and two-proportion z-test was conducted to compare the clinical efficacy of WX-081 and BDQ.

### 2.9 Calculation of QT-prolongation risk concentration for WX-081-M3

BDQ-M2 is responsible for the QT-prolongation risk and the concentration to induce ∼10 ms QT-prolongation (C_QT,WX-081−M3_) is 467 μg/L for TB patients (Anonymous 2012). Accordingly, the concentration of M3 with potential QT-prolongation risk (C_QT,WX-081−M3_) was determined for safety evaluation by correction of molecular weight and in vitro hERG IC_50_ (eqs. 5).

(5) $$C_{QT,WX-081-M3}=C_{QT,BDQ-M2}\times \frac{MW_{WX-081-M3}\times IC_{50,WX-081-M3}}{MW_{BDQ-M2}\times IC_{50,BDQ-M2}}$$

### 2.10 Software

PPK modeling and simulations were carried out in the software NONMEM (version 7.2) with the post-hoc FOCE-I algorithm, which was aided by functionalities implemented in Perl speaks NONMEM (PsN, version 5.0). The integrated software operating platform Pirana was used for documentation of the model development process. The screening of covariate was carried out under methods by using the stepwise covariate modeling (SCM) tool. The modeling dataset and simulated diagrams were processed by RStudio with R package (version 4.4.0). NCA was conducted using Phoenix WinNonlin (version 8.3.3).

## 3 Results

### 3.1 Subjects and pharmacokinetic data

A total of 1610 and 1580 PK observations were collected from 24 HVs and 48 TB patients for WX-081 and WX-081-M3, respectively. 0.86 % WX-081 and 2.71 % WX-081-M3 PK observations below LLOQ were excluded from the study.

### 3.2 Structural model development

**Fig. 1:**
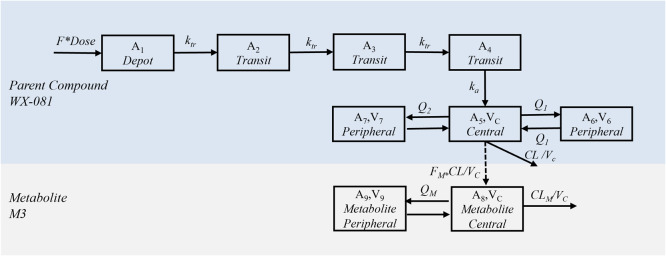
Schematic diagram of the structural model. The PK of the parent drug WX-081 was described by a three compartmental model for disposition together with three transit compartments for absorption and the PK of WX-081-M3 was depicted well with two compartments.

The structural PK model for WX-081 and WX-081-M3 was presented in Fig. 1. Three transit compartments between the depot and central compartment for WX-081 were included in the model. As a result, the absorption was quantified by the first-order rate absorption constant *k_a_* and transition rate *k_tr_*. The disposition of WX-081 was well described by three-compartments model and parameterized by apparent clearance (*CL/F)*, volume of distribution of central (*V_c_/F*), two peripheral compartments (*V_6_/F* and *V_7_/F*) and their respective inter-compartment clearance (*Q_1_/F* and *Q_2_/F*). The disposition of WX-081-M3 was well described by two-compartments model and parameterized by metabolite clearance (CL_M_/F*F_M_), volume of distribution of peripheral compartment (V_9_/F*F_M_) and inter-compartment clearance (Q_M_/F*F_M_).

### 3.3 Covariate model

After covariate analysis, baseline ALP was identified as a significant covariate on *CL_M_/F* (∆OFV: −8.4). Therefore, the final model was replaced by the covariate model and the estimates of final model parameters were presented in Table 2.

**Table 2:** Estimated PPK final model parameters with 500 bootstraps.

**Parameter**	**Unit**	**Theta**		**Omega**		**Shrinkage**
		**Estimate**	**RSE ( %)**	**IIV**	**RSE ( %)**	**( %)**
***CL/F***	L/hr	3.96 (3.11, 4.68)	7.7	0.40 (0.314, 0.458)	11	5.6
***V_c_/F***	L	13.1 (9.64, 18.8)	13.7	1.07 (0.842, 1.28)	10.9	31.6
***Q_1_/F***	L/hr	14.8 (13.2, 16.4)	6.6	0.386 (0.291, 0.465)	12.4	18.4
***Q_2_/F***	L/hr	4.66 (4.04, 5.59)	13.3	0.941 (0.603, 1.22)	10.7	7.4
***V_6_/F***	L	140 (111, 161)	4.7	NA	NA	NA
***V_7_/F***	L	1040 (598, 1948)	16.5	NA	NA	NA
***k_a_***	hr^-1^	1.49 (1.16, 2.00)	16.1	0.996 (0.775, 1.22)	13.8	27.8
***k_tr_***	hr^-1^	0.708 (0.647, 0.789)	3.5	NA	NA	NA
Q_M_/(F*FM)	L/hr	23.4 (11.6, 50.5)	12.8	NA	NA	NA
CL_M_/(F*FM)	L/hr	0.783 (0.529, 1.31)	9.1	0.399 (0.285, 0.517)	10.9	8.5
***F_M_***	%	2.35 (1.62, 4.02)	9	0.465 (0.381, 0.541)	8.6	0.5
V_9_/(F*FM)	L	92.9 (61.9, 173)	7.7	NA	NA	NA
***ALP on CL_M_***	–	0.44 (0.225, 0.755)	33.2	NA	NA	NA

*F*, bioavailability; *CL*, total clearance of WX-081; *V_c_*, volume of distribution for central compartment; *Q_1_ and Q_2_*, intercompartment clearance between central compartment and peripheral compartments; *V_6_* and *V_7_*, volume of distribution for peripheral compartment 1 and peripheral compartment 2; *k_a_,* first-order absorption rate constant; *k_tr_,* first-order transit rate constant; *Q_M_,* intercompartment clearance between metabolite central compartment and peripheral compartments; *CL_M_*, clearance of metabolite; *F_M_*, the fraction of WX-081 metabolized to WX-081-M3; *V_9_*, volume of distribution for metabolite peripheral compartment; *ALP on CL_M_*, the effect coefficient of ALP on metabolite clearance.

### 3.4 Model evaluation

**Fig. 2:**
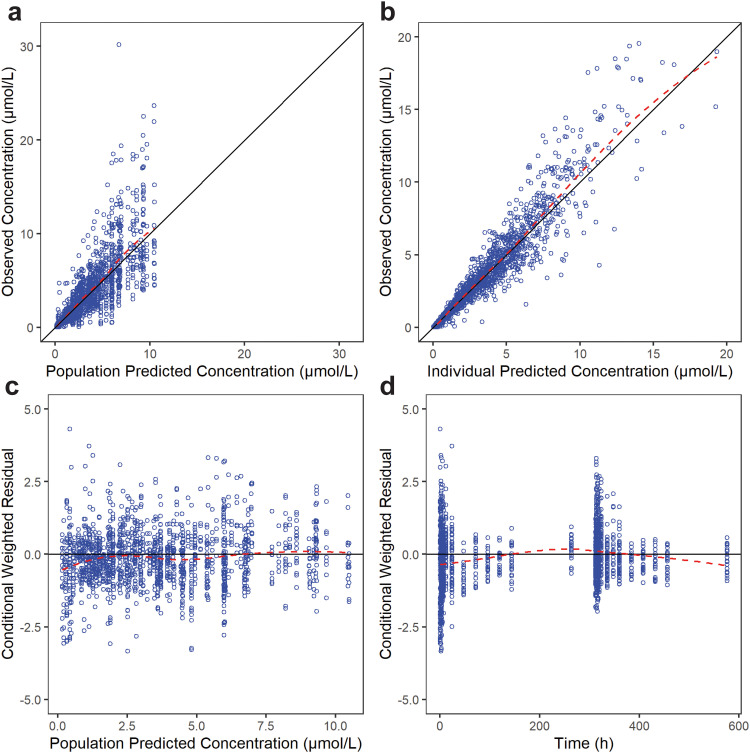
Goodness-of-fit plots for the final PPK model of WX-081. The red dotted line represents the fit of a LOESS regression through the data, with the blue circles representing the observed data.

**Fig. 3:**
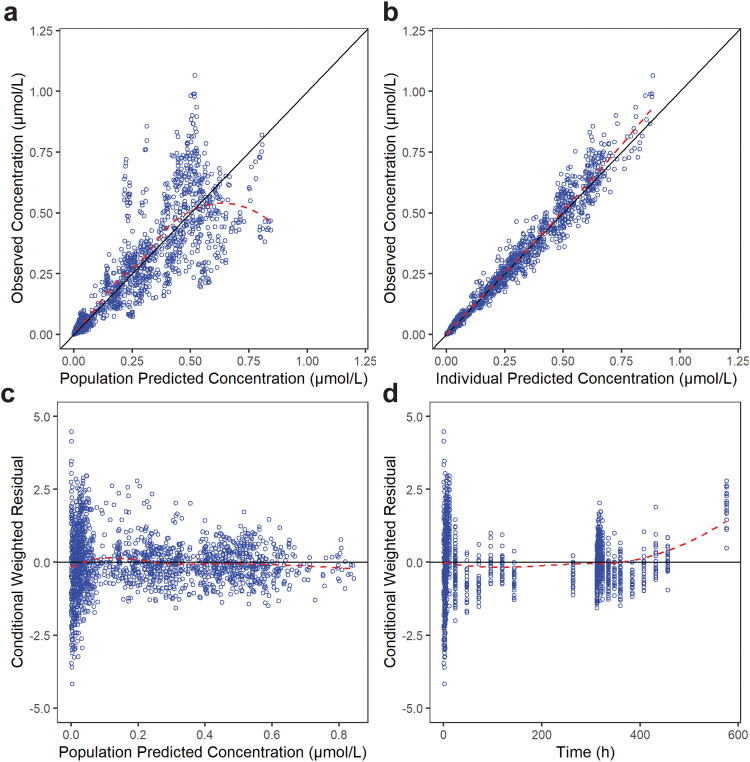
Goodness-of-fit plots for the final PPK model of WX-081-M3. The red dotted line represents the fit of a LOESS regression through the data, with the blue circles representing the observed data.

**Fig. 4:**
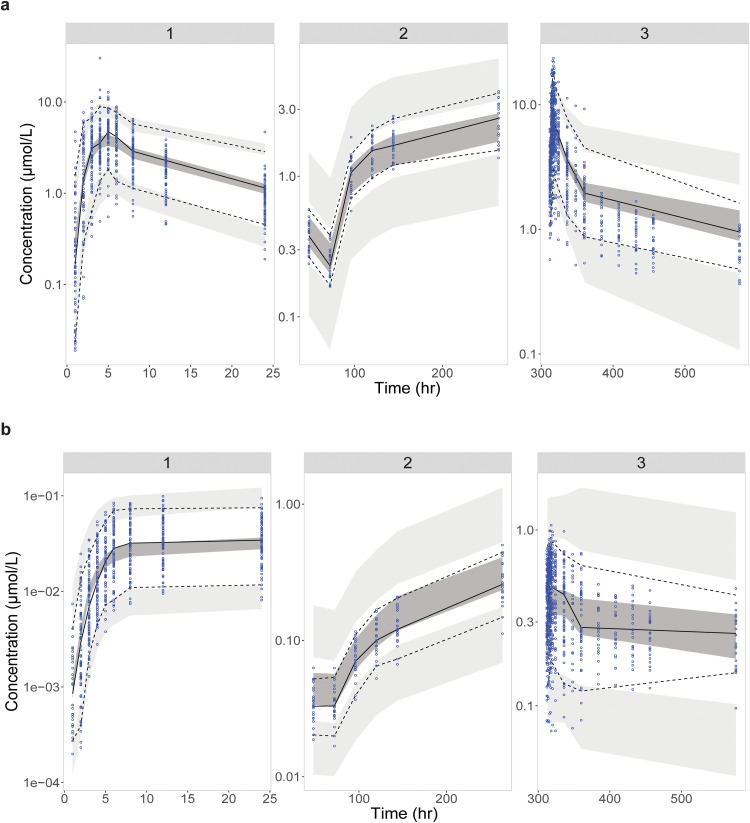
Visual predictive checks for the final population PK model for all data with WX-081 (above) and WX-081-M3 (below). The lower and upper dashed lines represent the 5th and 95th percentiles for the observed data. The solid line represents the 50th percentile for the observed data. The shaded areas represent the 90 % confidence intervals for the 5th, 50th, and 95th percentiles of the simulated data.

**Fig. 5:**
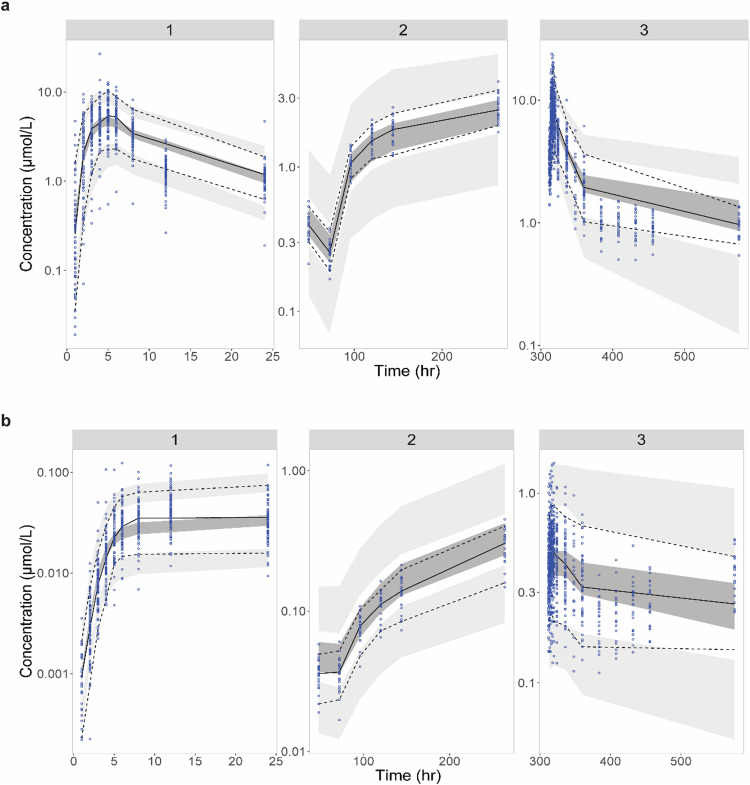
Prediction-corrected visual predictive checks for the final population PK model for all data with WX-081 (above) and WX-081-M3 (below). The lower and upper dashed lines represent the 5th and 95th percentiles for the observed data. The solid line represents the 50th percentile for the observed data. The shaded areas represent the 90 % confidence intervals for the 5th, 50th, and 95th percentiles of the simulated data.

GOF plots of the final model for WX-081 and WX-081-M3 were presented in Fig. 2, Fig. 3, respectively. For WX-081, plots of the observed versus IPRED or versus PRED showed that the data were scattered along the line of unity with low bias. As for WX-081-M3, although plots of the observed versus PRED showed bias at high concentration, the plots of the observed versus IPRED fit well. Plots of CWRES versus PRED and CWRES versus time after dose demonstrated that no apparent model misspecification at most concentration or time point, except for the last time point of WX-081-M3. The condition number (<1000) indicated that the model was not overparameterized and showed no collinearity. The estimated typical value of all parameters was within the range of 500 replicate bootstraps. The RSEs for the fixed and random effects were all < 20 % (Table 2). The VPCs presented in Fig. 4, Fig. 5 confirmed that the model well described the PK of both WX-081 and the WX-081-M3 (Bergstrand et al., 2011). Taken together, the model was stable and reliable.

### 3.5 E-R analysis

We conducted E-R analysis to compare the clinical efficacy between WX-081 and BDQ and to select a proper exposure metrics set to support dosage design. SCC was used as primary efficacy endpoint as did for BDQ (Diacon et al., 2009; Diacon et al., 2014). Firstly, Kaplan-Meier plot indicated that there is no significant difference between the clinical efficacy of WX-081 and BDQ (*P* = 0.7769 by log-rank test), as shown in Fig. 8a. Given that C_avg,ss_ was used to assess the efficacy of BDQ, C_avg,ss_ was also selected for ER evaluation for WX-081. E-R analysis suggested that C_avg,ss_ is indeed correlated with the efficacy of WX-081 (Fig. 8b). Furthermore, we tried to analyze the correlation between efficacy and other exposure parameters that are commonly used in the efficacy evaluation of antibiotic drugs to see if there is better parameter. We noticed that C_max,ss_ outperforms C_avg,ss_, while the correlation is weak for C_trough,ss_ (Fig. 8c and d). Consistently, AIC for ER model was 30.596, 31.457 and 31.724 for C_max,ss_, C_avg,ss_ and C_trough,ss_, respectively. Together, these data suggested that C_max,ss_ may serve as a better index for the evaluation of anti-TB efficacy. However, due to the limited sample size (*N* = 20), the conclusion is exploratory and warrant further validation in the Phase III study.

### 3.6 Model-based simulation

**Fig. 6:**
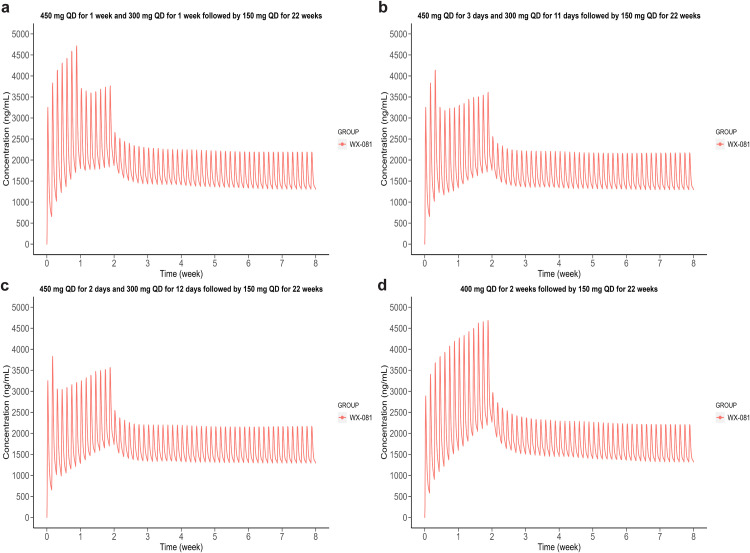
Simulation diagrams for WX-081 of (a) 450, 300 and 150 mg QD for 1, 1 and 22 weeks, (b) 450, 300 and 150 mg QD for 3 days, 11 days and 22 weeks, (c) 450, 300 and 150 mg QD for 2 days, 12 days and 22 weeks, (d) 400 and 150 mg QD for 2 and 22 weeks.

**Fig. 7:**
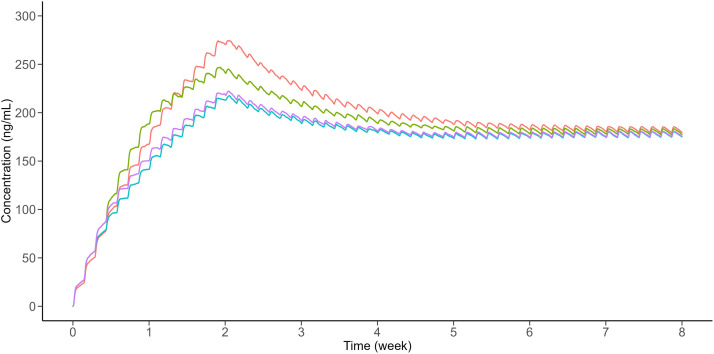
Simulation diagrams for WX-081-M3 of 450, 300 and 150 mg QD for 1, 1 and 22 weeks (green line), 450, 300 and 150 mg QD for 3 days, 11 days and 22 weeks (pink line),450, 300 and 150 mg QD for 2 days, 12 days and 22 weeks (blue line), 400 and 150 mg QD for 2 and 22 weeks (red line).

To select the proper dosage for phase III clinical trial, four regimens were simulated (Table 3). Dosage A, B and C were candidate regimens for phase III study, while the last regimen was used in phase II clinical trial. The dosage, C_avg,ss_ and C_max,WX-081−M3_ of four simulated regimens were listed in Table 3. The simulated concentration-time curves of WX-081 and WX-081-M3 were shown in Fig. 6, Fig. 7, respectively. As described in method section, the cut-off of correlated C_QT,WX-081−M3_ was set to 493 μg/L, respectively, according to the QT prolongation study of BDQ. As indicated by E-R analysis, the exposure of WX-081 under dosage D (phase II regimen) could be set as target for efficacy. The simulation results indicated that dosage A was the highest and extremely similar to that of regimen D with difference <1 %. Meanwhile, the C_max,WX-081−M3_ of dosage A was much lower than C_QT,WX-081−M3_. Generally, dosage A provide a similar exposure of WX-081 with flattened M3 C_max_, indicating a comparable efficacy and better safety. As a result, dosage A was recommended for phase III study.

**Table 3:** Summary of the dosage, simulated equivalent C_avg,ss_ and C_max,WX-081−M3_ under different regimen, presented as the median.

**Regimen**	**Dosage**	**C_avg,ss_ (μg/L)**	**C_max,WX-081−M3_ (μg/L)**
A	450 mg QD for 1 week and 300 mg QD for 1 week followed by 150 mg QD for 22 weeks	1612	247
B	450 mg QD for 3 days and 300 mg QD for 11 days followed by 150 mg QD for 22 weeks	1598	222
C	450 mg QD for 2 days and 300 mg QD for 12 days followed by 150 mg QD for 22 weeks	1595	217
Phase II	400 mg QD for 2 weeks followed by 150 mg QD for 22 weeks	1625	274

**Fig. 8:**
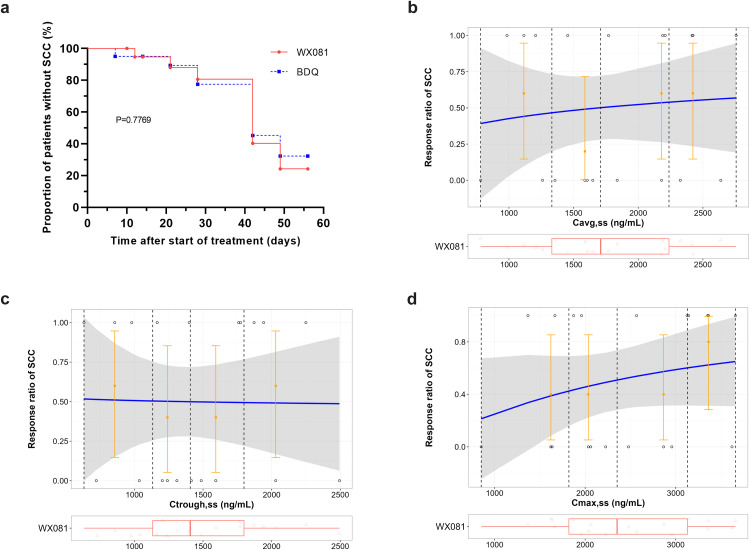
(a) Kaplan-Meier plot of head to head study for WX-081 and BDQ and (b-d) Exposure–response analysis of the correlation between SCC and C_avg,ss_ at day 56 as well C_trough,ss_ and C_max,ss_ for WX-081 in phase II. The blue line shows the estimated mean probability of SCC, while the shaded area represents 90 % confidence interval (CI). The circles represent the SCC data (0 = positive; 1 = negative) across the observed exposure range colored by exposure quartile. The error bars represent 90 % CI around the observed response rate in each exposure quartile. The box plot represents the distribution of observed exposure values.

## 4 Discussion

In this study, we constructed the first PPK model to characterize the PK profile for WX-081 and WX-081-M3 in healthy volunteers, DS-TB and MDR-TB patients. The model was utilized for simulation and in combination with E-R analysis to select proper dosage for phase III clinical studies regarding both efficacy and safety.

WX-081 shares similar but not identical PK characteristics and PPK models with BDQ. Dual zero-order input model was adopted to describe the dual peaks absorption of BDQ (Diacon et al., 2014). However, dual peaks were rarely observed for WX-081. Instead, multiple transit compartments model was used to fit its absorption. The CL/F of WX-081 (3.96 L/h) was slightly higher than that of BDQ (2.78 L/h). In addition, WX-081 was widely distributed with large V_c_/F (13.1 L), V_6_/F (140 L) and V_7_/F (1040 L). Similar with BDQ, the large distribution volume may be explained by its cationic amphiphilic nature with potential bind affinity to intracellular phospholipids and therefore a propensity to accumulate in tissues (McLeay et al., 2014).

Several covariates have been identified for BDQ. As reported by McLeay et al., the significant covariates include black race and subject status on CL/F and sex on V_c_/F (McLeay et al., 2014). Another study demonstrated that, in addition to age and race, time-varying covariates body weight and albumin also significantly affect the disposition of both BDQ and BDQ-M2 (Svensson et al., 2016). In a recently PPK study on the use of BDQ in Chinese MDR-TB patients, gamma-glutamyl transferase (GGT) and the single-nucleotide polymorphism (SNP) rs319952 in the AGBL4 gene were found to be significantly associated with the CL/F, while the abovementioned covariates are excluded (Zou et al., 2022). Despite the similarity in drug metabolism and PK profile between WX-081 and BDQ, the aforementioned BDQ covariates were not identified in our study. Instead, ALP was identified on CL_M_. According to preclinical studies, ALP was unlikely to be directly associated with the WX-081 metabolism. As one of the biomarkers for live function, ALP was speculated to indirectly reflect hepatic clearance of WX-081. The inconsistency may be partially ascribed to limited sample size or ethnic diversity, since only 72 Chinese subjects were included in our study. In addition, time-varying body weight and albumin data is absent in our study and their effect was not evaluated. At last, despite WX-081 exhibiting very high plasma protein binding (>99.0 %) based on non-clinical studies, no significant correlation was observed between albumin levels and WX-081 exposure parameters (Supplementary Figure S1–2). Also, the influence of disease state (defined as DS-TB and MDR-TB) was negligible (as shown in Supplementary Figures S3–4).

WX-081-M3 is responsible for QT prolongation risk with in vitro hERG inhibitory activity (Yao et al., 2022). As shown in our study, C_max,WX-081−M3_ is much lower than that of BDQ-M2 at the end of loading dose, which may be ascribed to the smaller AUC ratio between metabolite versus parent drug for WX-081 (0.08) compared to BDQ (0.31), respectively. Together with the similar in vitro hERG inhibitory activity as BDQ lower metabolite exposure indicated a better cardiac safety potential of WX-081, although the sample size of MDR-TB patients in phase II study is limited and may be insufficient to conclude the safety profiles of WX-081.

The approved regimen of BDQ includes a loading dose of 400 mg QD for 2 weeks followed by 200 mg TIW for 22 weeks. However, as part of the combination therapy for MDR-TB, other antibiotics are typically administered QD. Accordingly, the TIW regimen brings additional inconvenience for patients. Recently, a PPK study indicated that QD regimen of BDQ (200 mg QD for 2 months followed by 100 mg QD for 4 months) is able to provide similar BDQ exposure and C_max,BDQ−M2_ as approved regimen (Salinger et al., 2019). Thus, the daily administration of WX-081 may provide better compliance.

There are still several limitations in our study. Firstly, limited dataset and complex model structure lead to slight misfit for PPK evaluation. The terminal half-life (t_1/2_) of WX-081-M3 was so long that could not be fully captured by collected PK data within 24 days. Besides, available PK data, especially at terminal stage, may be insufficient for the complex PPK model that covers both WX-081 and WX-081-M3. As such, the IIV was slightly over-predicted before D3 administration and at the last observed time following D14 administration, especially for the metabolite (as shown in the VPCs). The over-prediction may be due to sparsely collected PK samples obtained during aforementioned time periods as well as relatively large variability. Besides, the loess regression trend in the GOF plot of WX-081-M3 suggested that terminal CWRES was slightly misfitted at the last sample time. As more PK data are collected in the on-going Phase III study, especially for terminal stage, we plan to further refine the model in the future. In addition, time-varying covariate such as body weight and albumin should be taken into consideration and the sequential data of these parameters are required in phase III study. Finally, given the risk of QT-prolongation and concomitant medication, the concentration-QT and drug interaction study is warranted in future studies.

## 5 Conclusions

In this study, we established the first PPK model that well described the PK characteristics of WX-081 and WX-081-M3 simultaneously. Combined with E-R analysis, the dosage of 450 mg QD for 1 week, 300 mg QD for 1 week followed by 150 mg QD for 22 weeks was recommended according to its efficacy and safety. Overall, our study provides a solid support for the clinical development of WX-081.

## Ethics approval

Trial protocols were approved by the Institutional Review Boards/Ethics Committees of the study sites.

## Consent to participate

All participants gave written informed consent before participating in the respective studies.

## Consent for publication

Not applicable.

## Funding

This work was supported by Bill &Melinda Gates Foundation (INV-007625).

## CRediT authorship contribution statement

**Weijie Kong:** Writing – original draft, Software, Methodology, Formal analysis. **Hao Liang:** Writing – review & editing, Methodology, Data curation. **Yi Zhang:** Writing – review & editing, Validation, Software, Methodology. **Lei Li:** Resources, Investigation. **Yongguo Li:** Resources, Investigation. **Xiaoyu Yan:** Writing – review & editing, Software, Investigation. **Dongyang Liu:** Writing – review & editing, Project administration, Investigation.

## Declaration of competing interest

Lei Li and Yongguo Li are employees of Shanghai Jiatan Biotech Ltd. All other authors declare no competing interests.

## Data Availability

The data that has been used is confidential.

## Appendix Supplementary materials

Image, application 1
