# Supplementary material for: Population pharmacokinetic and exposure-response study of a novel anti-tuberculosis drug to inform its dosage design in phase III clinical trial
Source: Eur J Pharm Sci. 2025 Sep 1;212:107160. doi: 10.1016/j.ejps.2025.107160 (PMC12357786; doi:10.1016/j.ejps.2025.107160)
Supplement: Supplementary file 1 [file mmc1.docx]

Population pharmacokinetic and exposure-response study of a novel anti-tuberculosis drug to inform its dosage design in phase III clinical trial

Weijie Kong^1,2#^, Hao Liang^1,2#^, Yi Zhang^1#^, Lei Li^3^, Yongguo Li^3^, Xiaoyu Yan^5*^, Dongyang Liu^1,4*^

^1^Drug Clinical Trial Center, Peking University Third Hospital, Beijing, China;

^2^Department of Nephrology, Peking University Third Hospital, Beijing, China;

^3^Shanghai Jiatan Biotech Ltd., a subsidiary of Guangzhou JOYO Pharma Ltd., Shanghai, China;

^4^Institute of Medical Innovation, Peking University Third Hospital, Beijing, China;

^5^School of Pharmacy, Faculty of Medicine, The Chinese University of Hong Kong, Hong Kong SAR, China

# These authors contributed equally

* Correspondence:

Dongyang Liu (liudongyang@vip.sina.com)

Xiaoyu Yan ([xiaoyuyan@cuhk.edu.hk](mailto:xiaoyuyan@cuhk.edu.hk))


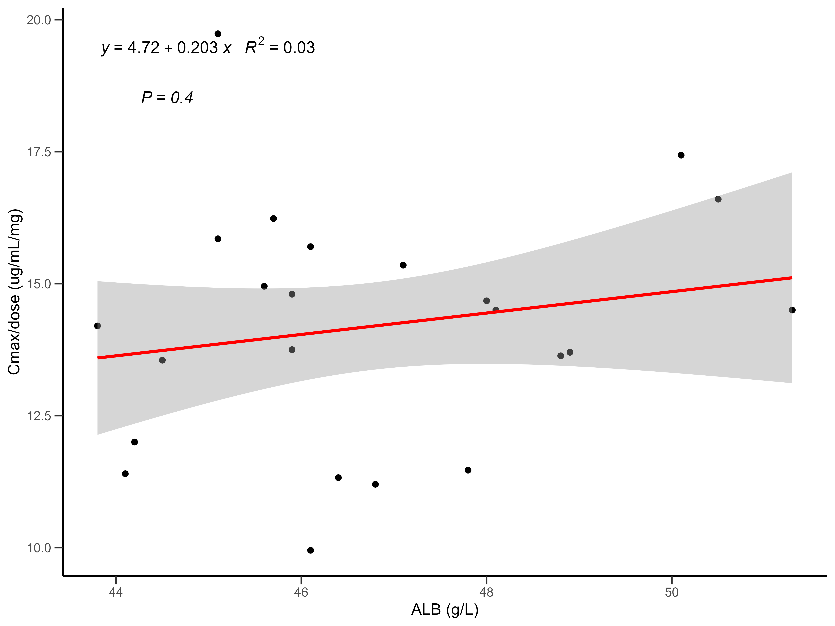


Supplementary Figure S1. Correlations of serum albumin concentration and dose normalized C_max_ (C_max_/dose) in HVs. The observed data was shown as black dot, while the predicted data was shown as red line (linear regression) and grey shadow (90% confidence interval).


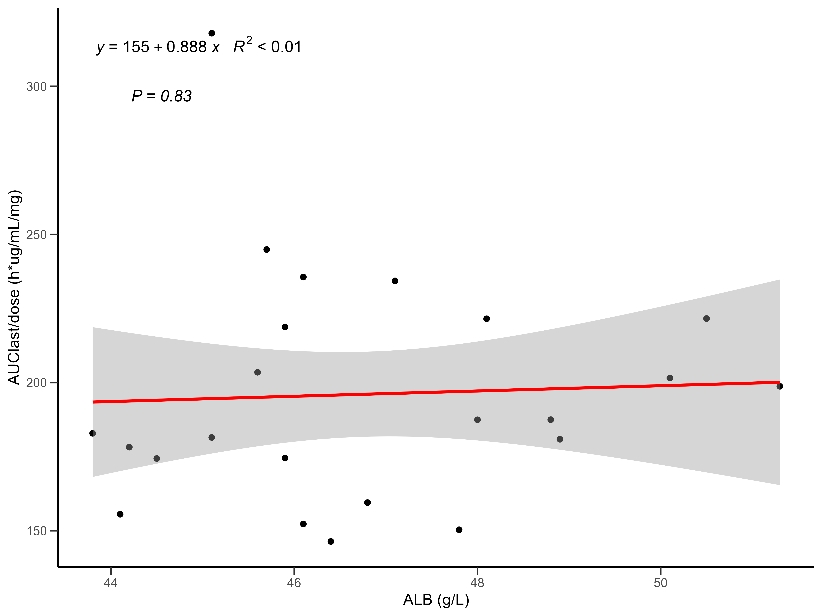


Supplementary Figure S2. Correlations of serum albumin concentration and dose normalized AUC_last_ (AUC_last_/dose) in HVs. The observed data was shown as black dot, while the predicted data was shown as red line (linear regression) and grey shadow (90% confidence interval).


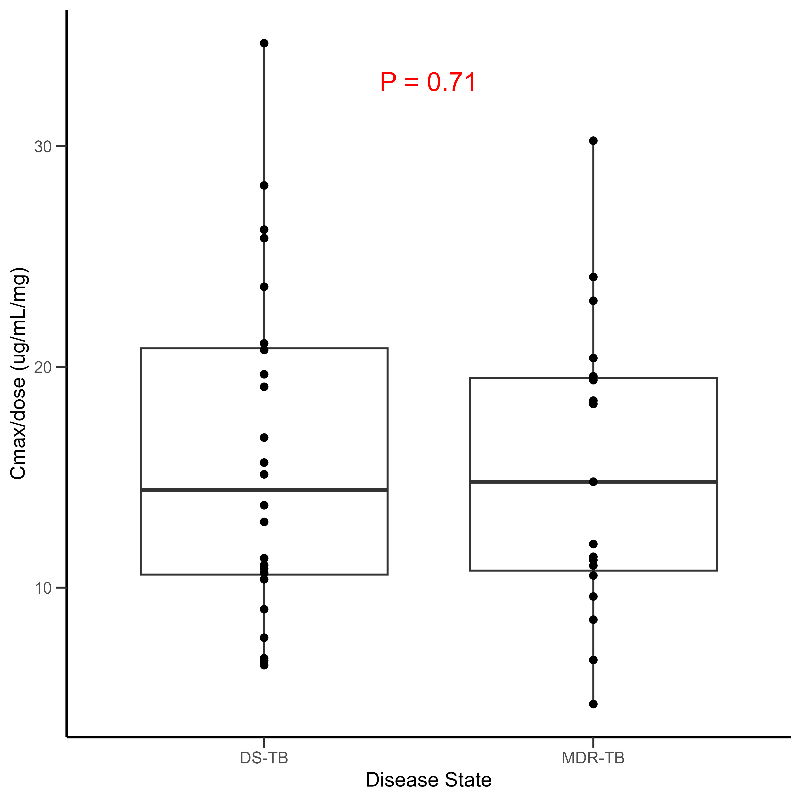


Supplementary Figure S3. Box-plot analysis of dose normalized C_max_ (C_max_/dose) in DS-TB and MDR-TB patients.


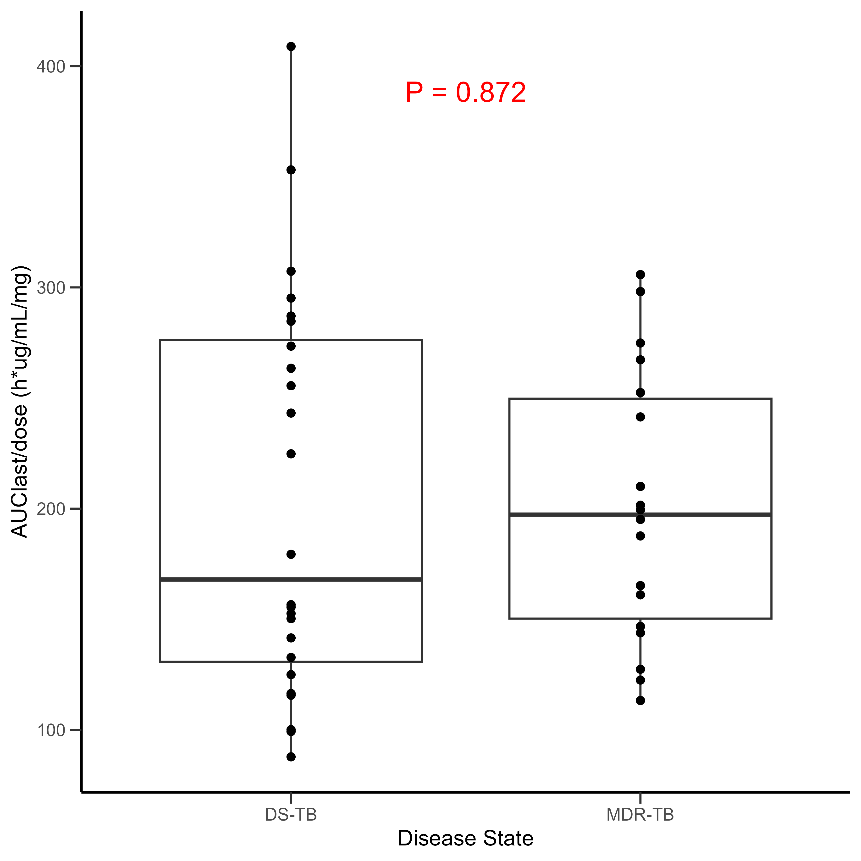


Supplementary Figure S4. Box-plot analysis of dose normalized AUC_last_ (AUC_last_/dose) in DS-TB and MDR-TB patients.
